# Endocrine and Cognitive Adaptations to Cope with Stress in Immature Common Marmosets (*Callithrix jacchus*): Sex and Age Matter

**DOI:** 10.3389/fpsyt.2015.00160

**Published:** 2015-11-30

**Authors:** Maria Bernardete Cordeiro de Sousa, Ana Cecília de Menezes Galvão, Carla Jéssica Rodrigues Sales, Dijenaide Chaves de Castro, Nicole Leite Galvão-Coelho

**Affiliations:** ^1^Brain Institute, Universidade Federal do Rio Grande do Norte, Natal, Brazil; ^2^Graduate Program in Psychobiology, Universidade Federal do Rio Grande do Norte, Natal, Brazil; ^3^Department of Physiology, Universidade Federal do Rio Grande do Norte, Natal, Brazil

**Keywords:** stress coping, cortisol, cognition, development, non-human primate model

## Abstract

Phenotypic sex differences in primates are associated with body differentiation during the early stages of life, expressed in both physiological and behavioral features. Hormones seem to play a pivotal role in creating a range of responses to meet environmental and social demands, resulting in better reactions to cope with challenges to survival and reproduction. Steroid hormones actively participate in neuroplasticity and steroids from both gonads and neurons seem to be involved in behavioral modulation in primates. Indirect evidence suggests the participation of sexual steroids in dimorphism of the stress response in common marmosets. This species is an important experimental model in psychiatry, and we found a dual profile for cortisol in the transition from juvenile to subadult, with females showing higher levels. Immature males and females at 6 and 9 months of age moved alone from the family group to a new cage, over a 21-day period, expressed distinct patterns of cortisol variation with respect to range and duration of response. Additional evidence showed that at 12 months of age, males and females buffered the hypothalamic–pituitary–adrenal axis during chronic stress. Moreover, chronic stressed juvenile marmoset males showed better cognitive performance in working memory tests and motivation when compared to those submitted to short-term stress living in family groups. Thus, as cortisol profile seems to be sexually dimorphic before adulthood, age and sex are critical variables to consider in approaches that require immature marmosets in their experimental protocols. Moreover, available cognitive tests should be scrutinized to allow better investigation of cognitive traits in this species.

## Introduction

During evolution, animals developed mechanisms to cope with challenging situations that are influenced by developmental phases and sex. In this context, steroid hormones seem to play a pivotal role in creating a range of responses to meet environmental and social demands, resulting in better reactions to cope with challenges to survival and reproduction. Their effects act on brain plasticity, changing neural circuitry constructed on a genetic, physical, and social environment basis, and involve synaptic sprout, spine growth, and trophic actions (1). Before the effects of steroids on the brain were known, the classification criteria of sexual dimorphism in males and females were based on sex chromosomes, gamete type, and morphology of the reproductive system. However, Phoenix et al. (2) demonstrated that female guinea pigs will display masculinized sexual behavior in adulthood when exposed to androgens during intrauterine development, indicating that the brain is susceptible to permanent hormonal changes in early life, a phenomenon they called the organizational effect. They also suggested that hormonal outcome may influence vertebrate morphology and behavior during other life stages, such as puberty and reproductive age (activational effects). These concepts were revised, and the organizational effects of hormones are currently considered to occur throughout life, and sex differences are expressed in conjunction with genetic (3, 4) and/or epigenetic mechanisms (5, 6), in addition to hormonal activity in the organs (7). These differences have repercussions on specific sex features that contribute to the sexual attributes of both sexes (8).

In humans, the biological basis of sex differences includes morphological, physiological, and behavioral changes, the last mechanism involving cognitive functions such as social and gender identity, preferred recreational activity, choice of social partners, as well as prevalence of genetic syndromes, cognitive performance, and susceptibility to drugs (8). This challenging multidimensional emotional and cognitive processing creates expectations in terms of new models to study psychiatric disorders (9–12).

The use of common marmosets as a model in studies involving emotional response to distress began around 20 years ago (13, 14), but has increased considerably this century, since their significant potential was revealed in terms of etiological, predictive, reactive, and functional validations to respond to human psychogenic and physical stressors (9, 15, 16). This species is also known for its sociobiology (17), with a social system based on dominance hierarchy, where reproduction is restricted to dominant pairs living in extended groups of 3–15 animals (18–21). Thus, behavioral and physiological studies using these animals are particularly enriching since biological data can be analyzed in the context of evolutionary pressures (ultimate and proximate). Approaches that investigate social isolation may challenge these social animals and trigger pathologies after long-term exposure that induces structural, behavioral, and cognitive changes (22). In this respect, sex and age are modulators of stress, which is expressed differently depending on the nature of the stressors (23).

On the other hand, differences in stress response across the lifespan are associated with different brain maturity levels in the specific areas implicated in neural organization of stress response and also involve experience in interpreting the harmfulness of the stressor (24). Clinical evidence for the dissimilarity in physical and mental illness in males and females after chronic exposure to stress should be considered in research involving human beings (25). Additionally, the evidence that stress has a different impact depending on critical periods of increased plasticity in the nervous system over the lifespan is a compelling factor for using animal models to study this issue (26–28).

Therefore, since adults exhibit dimorphic response to stress, where males respond to social isolation with a higher level of cortisol than females, we hypothesize that this difference may occur before adulthood. Thus, in this study, we investigated whether the cortisol profile of immature male and female common marmosets exhibits a dimorphic pattern, in addition to analyzing hormonal profiles in both undisturbed and challenging situations. We also investigated the influence of acute and chronic stress on the cognition of juvenile males.

## Materials and Methods

### Animals

Three experimental procedures using immature common marmoset (*Callithrix jacchus*) males and females were performed to investigate: (a) cortisol profile during natural development of male and female common marmosets living in their family groups (FGs) from birth until the onset of adulthood; (b) cortisol response to the paradigm of social isolation for 3 weeks in immature males and females at three age stages, according to the classification proposed by Leão et al. (29): juvenile I (6 months), juvenile II (9 months), and subadult (12 months); and (c) cortisol and cognitive performance after chronic social isolation for 4 months across the juvenile stage of males.

All animals used in the three experimental procedures were housed in the Laboratory for Advanced Primate Studies (LEAP, formerly Núcleo de Primatologia) at the Federal University of Rio Grande do Norte (UFRN), in cages under natural lighting, humidity, and temperature conditions. Animals received twice-a-day feedings that included seasonal fruits such as banana, papaya, melon, and mango, as well as potato and a protein potage containing milk, oats, egg, and bread. Water was available *ad ­libitum*. Animals were habituated to the presence of the researchers prior to the study, and a veterinarian provided health care throughout the experiment. The animals were treated in accordance with the criteria established by Brazilian Institute for the Environment and Renewable Resources (IBAMA) in Normative Instruction No. 169/2008 and Law No. 11.794, October 8, 2008, of the National Commission for Animal Care (CONCEA/Brazil). The LEAP follows international *ex situ* maintenance standards defined by the Animal Behavior Society and International Primatological Society. The studies were approved by the UFRN Ethics Committee for the use of animals (CEUA). The age, sex, and number of animals used in the three experimental protocols are shown in Table 1.

**Table 1:** **Number of animals used in the three experimental procedures by sex and/or age**.

Experiment	Males	Females
I – Animals living in their family groups monitored for 16 months from birth to early adulthood	4	6
II – Animals were moved from their families and monitored over a 3-month period at the following ages		
Juvenile I at 6 months	4	4
Juvenile II at 9 months	5	5
Subadult at 12 months	4	6
III – Animals were monitored for 5 months starting at juvenile I (from 7 to 11 months) under two conditions		
Living with their families for 5 months	5	–
Living with their families for 1 month and isolated for 4 months	5	–

### Experimental Procedures

#### Experiment I – Cortisol Levels Across Common Marmosets’ Developmental Ages

Ten common marmosets (*C. jacchus*), six females and four males, were followed from birth to 16 months (early adulthood), encompassing infancy, juvenile, and subadult stages. They lived in their FGs in individual outdoor cages and were submitted to fecal sampling once a week.

#### Experiment II – Cortisol Response to Social Isolation of Immature Males and Females

Thirteen immature common marmoset males and females were used in this experiment. The animals were divided according to the age classification proposed by Leão et al. (29) into juvenile I (>4–7; 6 months: four males and four females), juvenile II (>7–10 months; 9 months: five males and five females), and subadult (>10–15 months; 12 months: four males and six females) (Table 1). The study consisted of three phases: (i) Baseline phase (Bp) – animals were living in their FG, in cages measuring 3 m × 2 m × 2 m, with feces collected on two consecutive days to measure fecal cortisol before isolation; (ii) Isolation phase (Ip) – animals were socially isolated in a new cage measuring 2 m × 1 m × 1 m, for 21 days and fecal collections were performed on the first 2 days (Ip1: days 1 and 2) and last 2 days (Ip2: days 20 and 21) of the isolation period. During this phase, the animals were in auditory and olfactory, but not visual, contact with their conspecifics; (iii) Reunion phase (Rp) – animals were returned to their family cages and fecal samples were collected during the first 2 days after reunion. Food and water were freely available during all phases of the experiment.

#### Experiment III – Effects of Chronic Social Isolation on Cortisol and Cognition in Juvenile Males

##### Cortisol

Ten juvenile I males, aged 7 months, were followed until the age of 11 months (Table 1) to investigate cortisol response and cognitive impairment after chronic social isolation compared to the control group. Animals were divided into two groups: (i) FG: composed of five animals living for 5 months within their FG in cages measuring 3 m × 2 m × 2 m; (ii) Isolated group (IG): consisting of five animals that were followed during the first month living in their FGs and 4 months after social isolation in an individual cage, measuring 2 m × 1 m × 1 m. The duration of social isolation was in accordance with other studies on chronic stress in non-human primates (22, 30). During the experimental period, the animals in both situations had auditory and olfactory, but not visual, contact with other conspecifics. Food and water were available without restriction. For both groups, fecal samples were collected on alternate days in the first month, considered the Bp and feces were collected daily during the first week (seven samples per animal) of the four successive months (W2, W3, W4, W5a); only in the fifth month were feces collected in the last week (W5b).

##### Memory Tests

**Figure 1:**
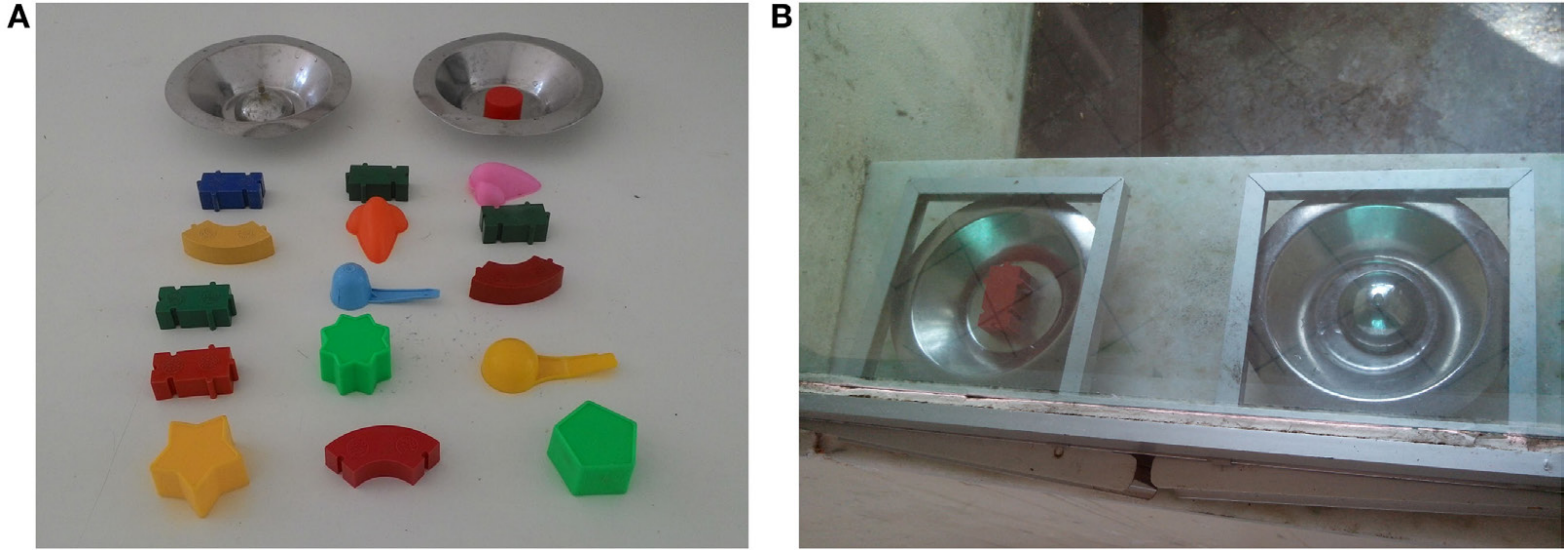
**(A)** Plates and **(B)** objects that were used for memory tests.

In Experiment III, two reverse learning memory tests – (1) object test and (2) box test – were used to assess working memory in all animals of the IG and FG groups. None of the animals had been submitted to cognitive tests. The object memory test involves memory discrimination and was adapted from Roberts and Wallis (31). Easy-to-handle three-dimensional plastic objects of different shapes and colors (Figure 1A) were used. During the tests, two different objects were placed on top of two plates presented to the animals through feed drawers located below the unidirectional visor, without animal interaction with the experimenter (Figure 1B). In the direct phase, the same object was paired with food reward and was repeatedly presented with different objects on the empty plate throughout this phase of the trials. After the animal learned the task, the next phase (reverse) began. In the reverse phase, the animals had to learn that the food was associated with the object that had been changed during the trials.

**Figure 2:**
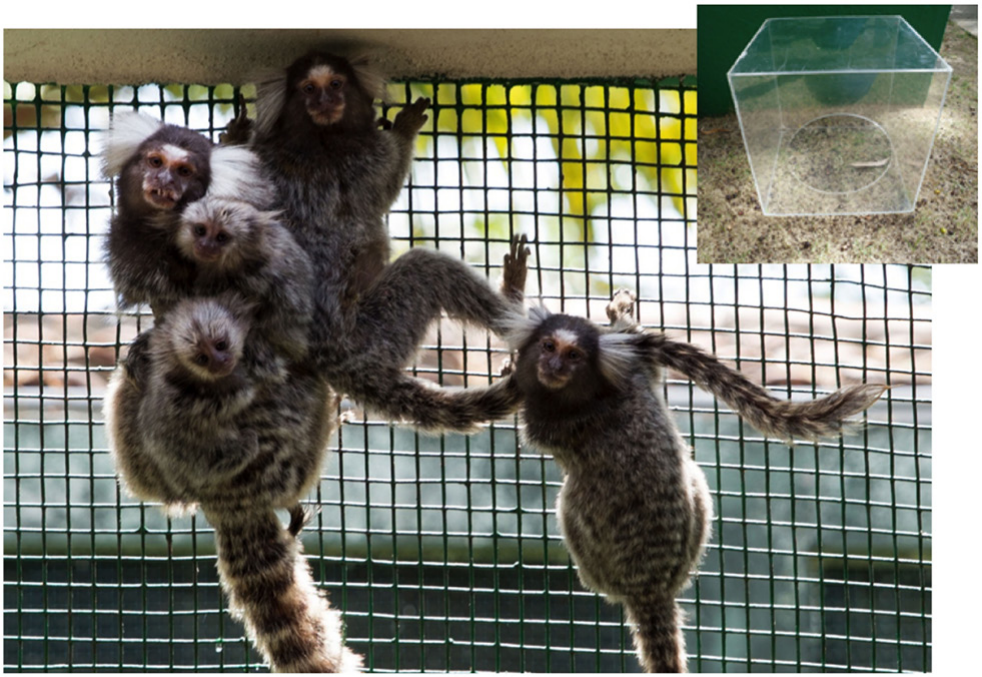
**The plexiglass container used for box memory tests at top right; common marmoset family living in the LEAP colony**.

The box memory test is a spatial memory test adapted from Murai et al. (32). A cuboid plexiglass box measuring 30 cm × 30 cm × 30 cm, with a 17-cm circular hole on one side, was used (Figure 2). This experimental apparatus was placed inside the cage and the experimenter observed the performance of the animals through a unidirectional visor in the front wall of the cage. During direct phase tests, the animal had to find the side of the box containing the hole, which was always facing upward, and obtain the reward. After the animal learned the task, the next phase (reverse) was initiated. In the reverse phase, the animal had to find the new side of the box where the opening was located and obtain the reward.

These tests were conducted inside the cage where the animals were housed. To test FG animals, they were rapidly separated, for 45 min at most, from their families into an adjoining cage divided by a solid wood plate. Before the beginning of the tests, in W5b, all animals were habituated to the experimental apparatus and FG animal acute family separation (10–15 min) was necessary during this protocol. All animals were randomly handled six times, three times in the morning and three times in the afternoon, over 3 days within 1 week.

After the 5-month period, memory tests were applied to animals from both IG and FG groups over the next 3 days. Object tests were conducted in the morning (between 7:30 and 9:00 a.m.) and box tests in the afternoon (between 2:00 and 4:00 p.m.) on the same day, always 2 h after the animals were fed. Trial duration was at most 3 min and the delay between trials was approximately 5 s. The animals were submitted to 15 trials for each of the direct and reverse phases of both object and box memory tests. In both phases of the two tests, it was expected that the animal would learn where the food reward was. When the animal achieved six consecutive correct responses, it was considered to have learned the task, which was then interrupted. In order to properly execute the reverse-phase task, the animal had to inhibit the learning of the direct phase. The test results were calculated as learning indices (H = hits; E = errors) and motivation index (Li = lack of interest).

### Fecal Cortisol Measurement

In Experiment I, fecal samples were taken in the morning and afternoon, since diurnal variation of cortisol during development must be monitored. In the other two experiments, cortisol was quantified from fecal samples collected at similar times, between 6:00 and 9:00 a.m., to avoid circadian variation in hormone profile (33). In all experiments, the observer was aware of defecation through a unidirectional visor. When the animal defecated samples were collected, identified, and stored in small glass tubes at −4°C until hydrolysis and solvolysis procedures for cortisol extraction and measurements.

For Experiment I, fecal collection was possible only when infants were 1 month of age or older. All processing of cortisol extraction and measurements using immune-enzymatic (ELISA) competitive assays were according to Sousa and Ziegler (34). Intra and interassay coefficients of variation were 1.97 and 3.39%; 23.40 and 29.40%; and 1.23 and 29.35% for Experiment I, II, and III, respectively.

### Statistical Analysis

#### Experiment I

For the study of cortisol during development for the same individual across the lifespan, repetitive measures are observed. Moreover, the time of day and observations of a same animal are correlated. In this type of analysis, it is important that the models include possible dependencies among observations of the same individual. The changes in cortisol at different age phases are an example of these variations, since the focus is on evaluating hormonal changes across the phases. Therefore, it is necessary to simultaneously fit the model to the structure of the general mean as well as both intra and interindividual variability.

Logarithmic transformation of the hormonal data was performed to obtain good model fit and achieve normalization and variability constancy of the residues, demonstrating suitable model fit. After the significance of the age factor was detected, Tukey’s test for multiple comparisons among means of the age stages was used.

#### Experiment II

After normalization of hormonal data by logarithmic transformation, the ANOVA parametric test for repeated measures and Fisher’s *post hoc* test were applied to investigate cortisol variations using sex and age as independent variables and phases of the study as dependent variable. An outlier value of four standard deviations above the mean was excluded from one male aged 6 months.

#### Experiment III

After normalization of hormonal data by logarithmic transformation, the ANOVA parametric test for repeated measures and Fisher’s *post hoc* were used to investigate cortisol variations between the groups (IG and FG) and independent variables through the phases (Bp, W2, W3, W4, W5a, and W5b). For memory tests, hit rates, errors, and lack of interest were transformed into continuous data for use in the non-parametric Mann–Whitney test to investigate the difference between FG and IG in each phase (forward and reverse) of the test separately. The Spearman correlation test was performed using mean cortisol levels in W5b versus the sum of the indices for learning or motivation. An outlier value of four standard deviations above the mean was excluded from one animal in W3.

The significance level for all variables in the three experiments was set at *p* ≤ 0.05. A near-significant trend when *p* was between 0.05 < *p* ≤ 0.07 was considered.

## Results

### Experiment I – Cortisol Levels Across Common Marmoset Developmental Ages

**Figure 3:**
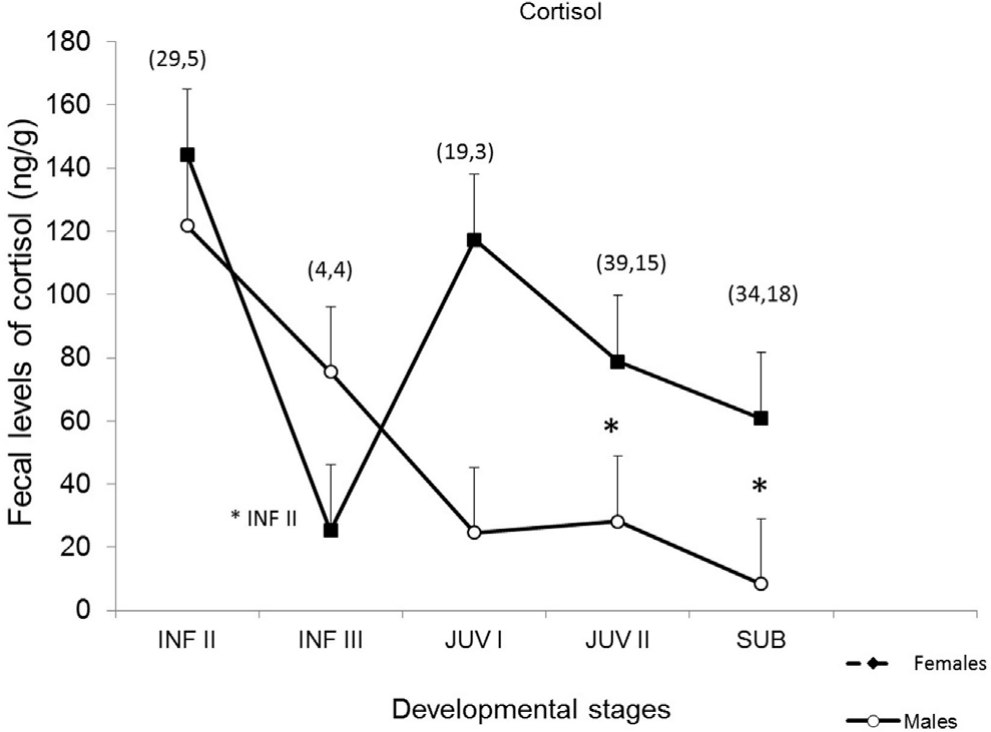
**Fecal cortisol in male (*n* = 4) and female (*n* = 6) common marmosets across developmental phases (INF I, II, III: infantile I, infantile II, infantile III; JUV I, II: juvenile I, juvenile II; SUB: subadult) [based on Ref. (29)]**. Tukey’s test was performed, *p* < 0.05. The symbol “* INF II” indicates significant statistical difference between ages INF II and INF III in females; The symbol “*” indicates significant statistical differences between males and females at juvenile II and subadult ages.

The developing fluctuation patterns for fecal cortisol in males (*n* = 4) and females (*n* = 6) are shown in Figure 3. As previously stated, fecal collection was possible only after animals reached the infantile II stage and includes samples from the morning and afternoon. Thus, it is expected that cortisol levels reach higher levels than those observed for protocols where fecal collections include only samples from the morning, since cortisol excretion is significantly higher in the afternoon (34).

Mean total cortisol values are higher during the infantile II stage (>1–3 months) when compared to successive age-related development stages (ANOVA, *p* < 0.05) and were different for both sexes at juvenile II and subadult, with a significant decrease during the infantile III (>3–4 months) stage, which was statistically significant only for females when compared to the previous phase. From the juvenile stage to the end of the study, when animals were classified as subadults (>15–16 months), age-related cortisol excretion in females was higher than that of males at juvenile II (Tukey’s test *p* = 0.036) and subadult (*p* = 0.004) ages.

### Experiment II – Cortisol Response to Social Isolation of Immature Males and Females

**Figure 4:**
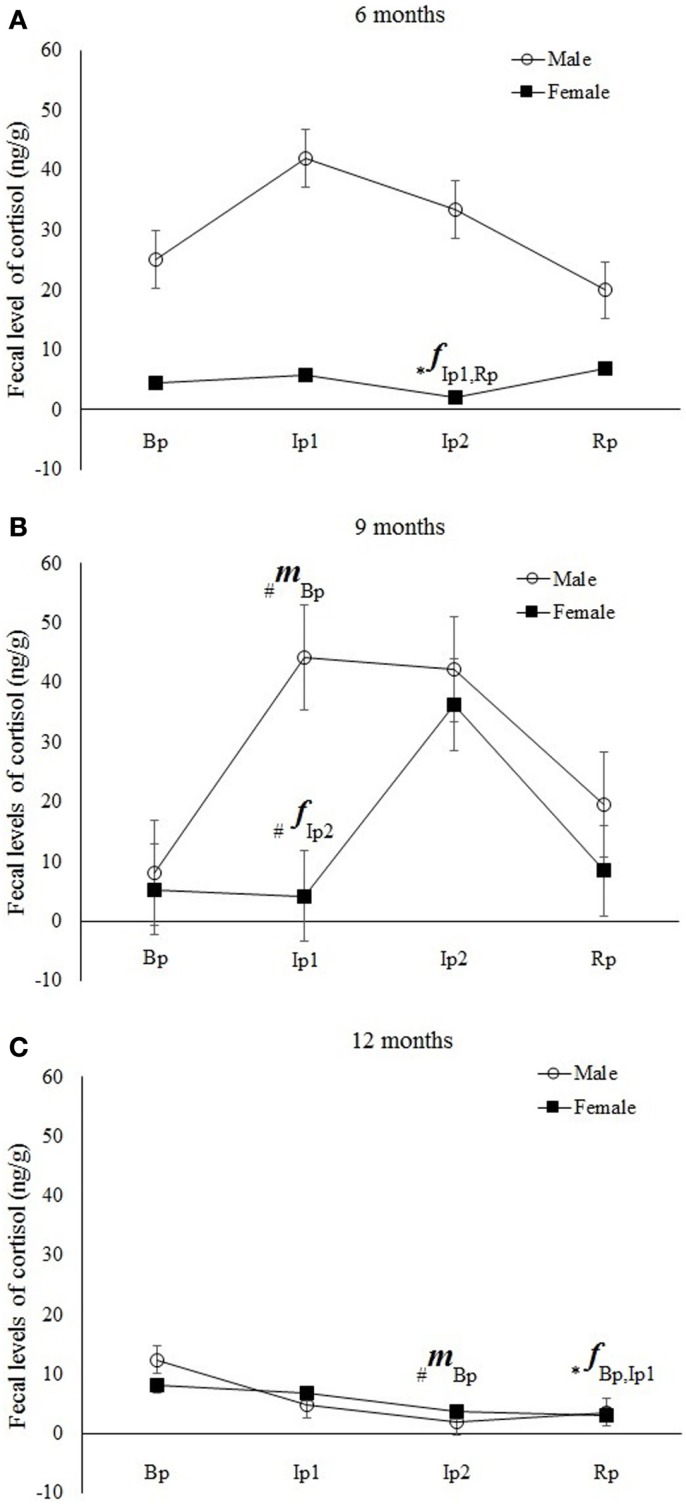
**Mean (±SE) of fecal cortisol levels (ng/g) in *Callithrix jacchus* in the Experiment II (Bp = baseline phase; Ip1 = initial isolation phase; Ip2 = late isolation phase; and Rp = reunion phase) at (A) 6 months, (B) 9 months, and (C) 12 months of age**. A multivariate ANOVA test for repeated measures was performed with the Fisher posttest, *p* < 0.05. The symbol “*” indicates significant statistical differences and “#” a statistical tendency to differences in cortisol levels between the respective study phase and phases represented beside the symbol. The letters “*f*” and “*m*” indicate females and males, respectively.

In Experiment II, we used immature animals at juvenile I (*n* = 8/4 males), juvenile II (*n* = 10/5 males), and subadult (*n* = 10/4 males) development stages living in their FGs, isolated in cages and reunited with their family. A significant interaction among sex, age, and phases was found (ANOVA; Sex × Ages × Phases: *F* = 2.20, *p* = 0.04) as shown in Figure 4.

Six-month-old males showed no variations in fecal cortisol levels across the phases (*post hoc* Fisher; Bp*Ip1, *p* = 0.8; Bp*Ip2, *p* = 0.7; Bp*Rp, *p* = 0.3; Ip1*Ip2, *p* = 0.9; Ip1*Rp, *p* = 0.2; Ip2*Rp, *p* = 0.19) (Figure 4). Nine-month-old males exhibited a statistically significant increase in cortisol levels during the initial isolation phase (Ip1) with respect to baseline (Bp) (*post hoc* Fisher; Bp*Ip1, *p* = 0.04) and a statistical tendency to reduction in the Rp with respect to Ip1 (*post hoc* Fisher; Bp*Ip2, *p* = 0.13; Bp*Rp, *p* = 0.8; Ip1*Ip2, *p* = 0.60; Ip1*Rp, *p* = 0.07; Ip2*Rp, *p* = 0.18) (Figure 4). Subadults (12 months) showed a statistical tendency to reduction in cortisol levels in the final isolation phase (Ip2) compared with baseline (Bp) (*post hoc* Fisher; Bp*Ip1, *p* = 0.13; Bp*Ip2, *p* = 0.06; Bp*Rp, *p* = 0.34; Ip1*Ip2, *p* = 0.75; Ip1*Rp, *p* = 0.5; Ip2*Rp, *p* = 0.3) (Figure 4).

For females, six-month-old animals showed a significant reduction in fecal cortisol levels in Ip2 with respect to Ip1 and Rp (*Post hoc* Fisher; Bp*Ip1, *p* = 0.59; Bp*Ip2, *p* = 0.1; Bp*Rp, *p* = 0.3; Ip1*Ip2, *p* = 0.03; Ip1*Rp, *p* = 0.73; Ip2*Rp, *p* = 0.01). Nine-month-old females exhibited a statistical tendency to increasing cortisol levels during the final isolation phase (Ip2) with respect to the initial isolation phase (Ip1) (*Post hoc* Fisher; Bp*Ip1, *p* = 0.6; Bp*Ip2, *p* = 0.15; Bp*Rp, *p* = 0.9; Ip1*Ip2, *p* = 0.06; Ip1*Rp, *p* = 0.6; Ip2*Rp, *p* = 0.1). Subadult (12 months) females showed a significant reduction in cortisol levels in the Rp compared with baseline (Bp) and Ip1 (*Post hoc* Fisher; Bp*Ip1, *p* = 0.65; Bp*Ip2, *p* = 0.13; Bp* Rp, *p* = 0.01; Ip1*Ip2, *p* = 0.2; Ip1*Rp, *p* = 0.04; Ip2*Rp, *p* = 0.3) (Figure 4).

Considering the entire set of results, it is noteworthy that young animals exhibit distinct cortisol response patterns in relation to sex and age, although no typical pattern could be characterized. In general, more immature males and females (juvenile I and II) exhibited different cortisol levels during the isolation phase with different intensities and temporalities, whereas subadult males and females showed a similar reduction in cortisol levels across experimental phases, as demonstrated by significant results and trends observed after statistical analysis.

### Experiment III – Effects of Chronic Social Isolation on Cortisol and Cognition in Juvenile Males

#### Cortisol

**Figure 5:**
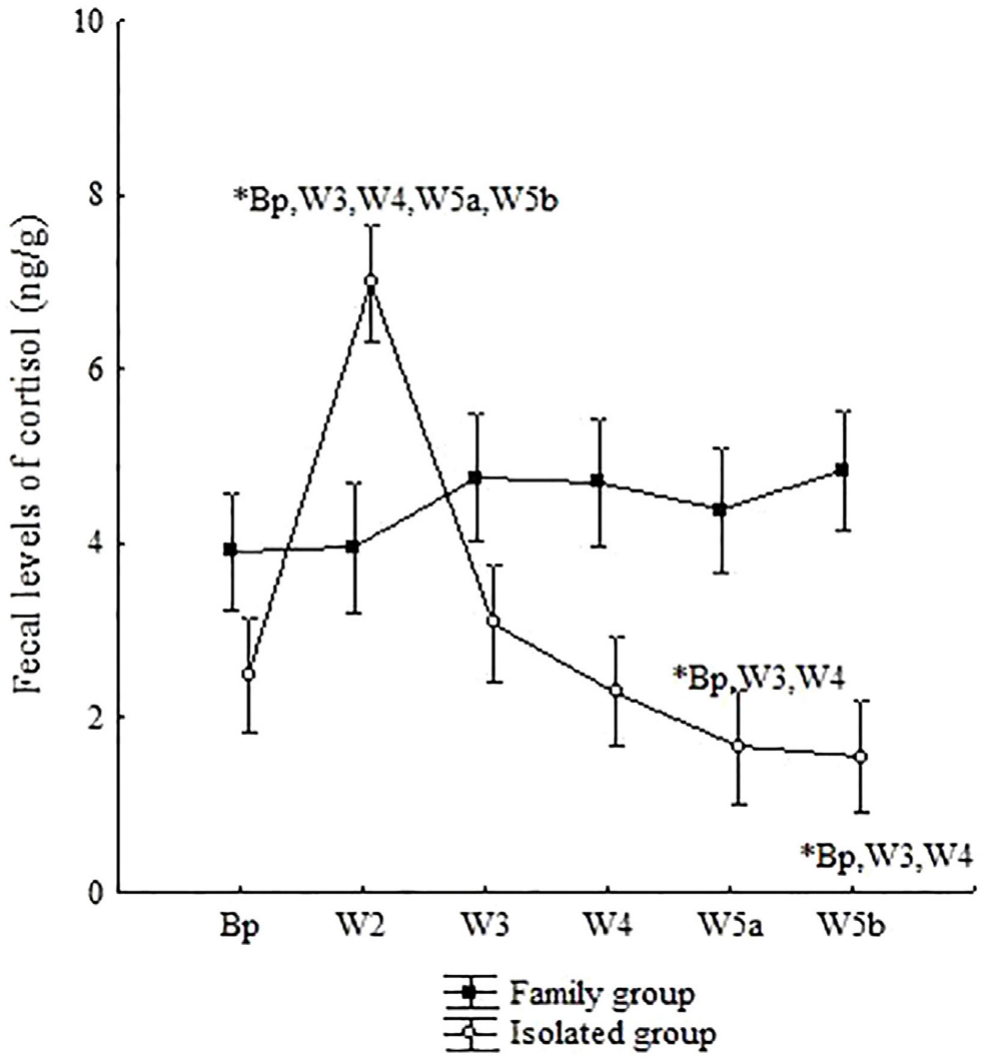
**Mean (±SE) of fecal cortisol levels (ng/g) in common marmoset males in Experiment III, living in family groups (FG) or under chronic social isolation for 4 months (IG)**. Bp = baseline phase; W2 = first week of second month; W3 = first week of third month; W4 = first week of fourth month; W5a = first week of fifth month; and W5b = last week of fifth month of isolation. A multivariate ANOVA test for repeated measures was performed using the Fisher posttest, *p* < 0.05. The symbol “*” indicates a significant difference in cortisol levels between the respective study phase and those represented beside the symbol.

Cortisol levels in the animals living with their families (FG) showed no statistically significant variations during the study (ANOVA, Fisher *post hoc –* W2*Bp: *p* = 0.96; W2*W3: *p* = 0.94; W2*W4: *p* = 0.8; W2*W5a: *p* = 0.82; W2*W5b: *p* = 0.48; W3*Bp: *p* = 0.91; W3*W4: *p* = 0.86; W3*W5a: *p* = 0.8; W3*W5b: *p* = 0.53; W4*Bp: *p* = 0.77; W4*W5a: *p* = 0.98; W4*W5b: *p* = 0.65; W5a*Bp: *p* = 0.78; W5a*W5b: *p* = 0.64; W5b*Bp: *p* = 0.44) (Figure 5).

For the animals that were separated from their FG, a sharp rise in cortisol was observed in W2, with higher hormone levels in this phase when compared with all other phases of the study (ANOVA, Fisher *post hoc* – W2*Bp: *p* = 0.001; W2*W3: *p* = 0.001; W2*W4: *p* = 0.001; W2*W5a: *p* = 0.001; W2*W5b: *p* = 0.001). The decreasing cortisol profile during the isolation period was characterized by a gradual reduction across the phases, with the lowest concentrations in W5a and W5b in relation to baseline levels (Fisher *post hoc* – W3*Bp: *p* = 0.51; W3*W4: *p* = 0.39; W3*W5a: *p* = 0.001; W3*W5b: *p* = 0.004; W4*Bp: *p* = 0.83; W4*W5a: *p* = 0.005; W4*W5b: *p* = 0.04; W5a*Bp: *p* = 0.003; W5a*W5b: *p* = 0.40; W5b*Bp: *p* = 0.02) (Figure 5).

In short, it is interesting to observe that marmosets under chronic social isolation (IG) showed a different cortisol pattern in relation to that of the FG, ranging from an increase at the beginning of the isolation period to decreased levels in the final isolation phases.

#### Memory Tests

##### Object Memory Tests

**Figure 6:**
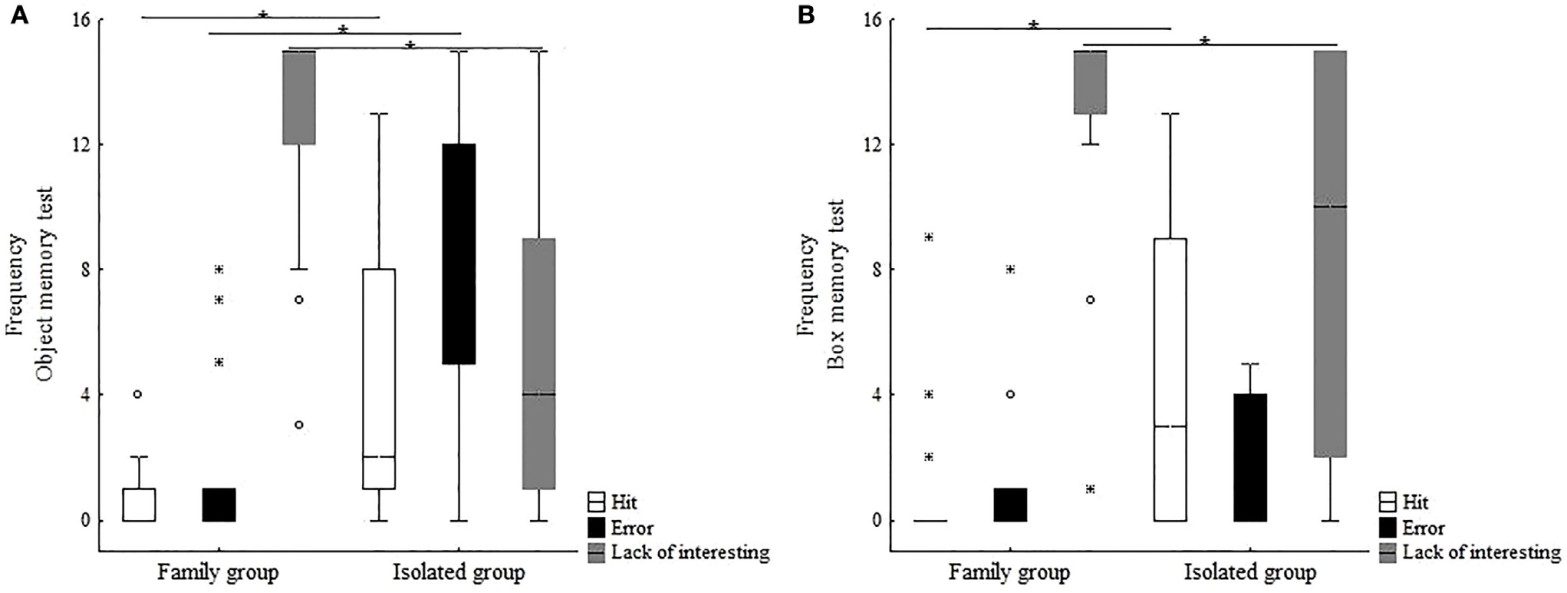
**Indexes of hits, errors, and lack of interest investigated in immature common marmosets at subadult age living with their families (FG) or in chronic social isolation (IG): (A) object memory tests and (B) box memory tests**.

The performance of FG and IG animals differed in the object memory tests, with higher hit (H) (Mann–Whitney *U* = 45, *p* = 0.004) and error (E) (Mann–Whitney *U* = 36, *p* = 0.001) rates for IG, whereas FG exhibited higher lack of interest (Li) levels than IG (Mann–Whitney *U* = 30, *p* = 0.0001) (Figure 6A). Since none of the animals in either group was able to learn the direct task, the reverse phase was not presented.

For both FG and IG groups, negative correlations between cortisol levels in the last week of study (W5b) and AI (Spearman correlation, FG: *r*_s_ = −0.9; IG: *r*_s_ = −0.9) were found.

##### Box Memory Test

When the animals were resubmitted to the direct phase of the box memory test (Figure 6B), significantly higher H rates were observed for IG (Mann–Whitney *U* = 57, *p* = 0.021), whereas FG displayed higher Li (Mann–Whitney *U* = 65, *p* = 0.05). No significant intergroup difference was found in the number of E (Mann–Whitney *U* = 103, *p* = 0.713). Only 3 of the 10 IG animals learned the direct task and started the reverse phase. However, none learned the reverse task in the time period used.

Again, for both groups (FG and IG), negative correlations between cortisol levels in the last week of study (W5b) and AI (Spearman correlation, FG = *r*_s_ = −0.97; IG = *r*_s_ = −0.89) were observed. Additionally, for IG, a negative correlation between cortisol and hits (Spearman correlation: *r*_s_ = −0.9) and a positive correlation with errors (Spearman correlation: *r*_s_ = 0.9) were recorded.

## Discussion

In this study, we investigated the age and sex of common marmosets (*C. jacchus*), a non-human primate widely used as an experimental model to obtain new information to better understand basal cortisol profile and postnatal stress exposure during developmental stages. We found a different profile for cortisol during marmoset development, with females showing low levels at infantile III, and higher at both juvenile II and subadult ages when compared to that of males. Immature males and females at 6 and 9 months of age moved alone from the FG to a new cage, over a 21-day period, expressed distinct patterns of cortisol variation with respect to range and duration of response but during juvenile to subadult transition (at 12 months) both males and females buffered similarly the hypothalamic–pituitary–adrenal (HPA) axis during chronic stress. Moreover, during juvenile to subadult transition, the patterns of cortisol reduction were maintained in males under social isolation for 4 months and were associated to better cognitive performance. The results for memory testing showed that acute and chronic stress impaired working memory and marmosets were more motivated to perform these tests when under chronic isolation.

Common marmosets, like other New World primates, exhibit extremely high cortisol levels and adaptations of the adrenal reticular zone, in terms of morphology and functionality that are different in males and females (35, 36). These authors demonstrated that the adrenals of neonatal marmosets exhibit functional secretion of the C19 steroid, as observed in humans, but adult males do not develop a functional reticular zone (35). They also found that anovulatory subordinates and ovariectomized common marmoset females displayed elevated cytochrome b5 (cytb5) expression in the adrenal reticular zone, generating significant ACTH-responsive DHEA production, suggesting both gonadal and sex differences in adrenocortical regulation.

Experiment I sought to characterize the cortisol production pattern in common marmoset males and females, from birth to early adulthood at the intermediate ages between infancy and adulthood, living in FGs in an undisturbed condition. The main findings included similar cortisol levels in infantile II, a significant decrease in cortisol production during infantile III, and a further increase that occurs only in females, with males maintaining low cortisol levels in juvenile II to subadulthood. In a study where common marmosets were also followed from birth to adulthood to investigate pituitary–adrenal basal function (27), ACTH and cortisol plasma levels were elevated in newborns and infants (4 weeks) compared to 2-month-old infants, juveniles (6 months), subadults (12 months), and adults, but no intersex differences were observed. These general age differences are in accordance with the current study, but male and female cortisol differences across development were found in the present study and might have resulted from the fact that these authors did not collect samples from common marmosets at 4 months of age, thereby missing these transient changes at infantile III age. This result suggests that differences in the functioning between adrenals of male and female common marmosets might start early in life, during the transition between infantile and juvenile stages, and could be a consequence of different female needs for developing physiological systems, including differentiating the behavioral expression of their sexual strategies and/or different demands for immediate reproductive maturation. Gonadal activation in common marmoset males starts at around 4 months, i.e., around the infantile III stage, and this decrease observed only for females may be due to puberty steroid production and puberty changes at 6–7 months (37), sexual maturity occurring at approximately 16 months. For females, estradiol peaks start at around 6 months, and progesterone increases at about 13 months, with sexual maturity occurring at approximately 16 months (38, 39). However, studies in which females were paired with adult males showed that they were able to ovulate at the age of 10 months (40). Sex steroid production by these same animals was studied by Castro (41), who found that sex hormone secretion starts at around 5 months and puberty (sex steroid production in amounts close to those of adults) at 5–7 months, for both sexes, suggesting a common range for gonadal maturation in males and females. Thus, these results might be indicating a surge of an early dimorphic pattern of cortisol production in marmosets that is also expressed in adults, when the HPA axis of males is more responsible to separation than that of females, probably associated with different reproductive strategies (42).

In Experiment II, immature males and females in juvenile I and II and subadult stages were separated from FGs, and after reunion showed differences in cortisol profile. Common marmoset males in juvenile I, aged 6 months, exhibited a changeable pattern of cortisol over the course of the study (21 days). Females also showed a changeable profile of cortisol with a slight reduction in the last phase of isolation when compared to previous isolation and reunion phases. Juvenile II males and females, aged 9 months, also showed changeable cortisol profile but characterized by increased and distinct patterns of temporality. These findings seem to be compatible with development of the mechanisms that occur at this age related to maturation and integration of both HPA and HPG axes. Some studies suggest that maturation of the HPG axis is a major factor for the emergence of sexual dimorphism in the HPA axis (43). Thus, the sexual immaturity of animals observed in this study might have produced the variable cortisol profile. Alternatively, the small sample size may also have contributed to this variance. Assessing the temporal dynamics of stress response is important in determining the costs and benefits of this reaction (44). The return to baseline is an important component of the stress response because the deactivation systems reduce the risk of diseases associated with chronic stress (45, 46). Adjustments to feedback control systems during ontogenesis contribute to calibrating the stress response, making it more adaptive to that particular situation (47). In a related species of marmoset (*Callithrix geoffroy*), in which an acute stress protocol (8 h of isolation from the FG) was used for males and females at 6, 12, and 18 months (48), no significant sex differences were found in cortisol at any age. However, they observed age-related differences in cortisol variation over the 8 h, with the highest, intermediate, and lowest reactivity at 6, 12, and 18 months, respectively. Nonetheless, the evolution of cortisol profile for all animals was similar, with an increasing pattern from the first 2–4 h after isolation that remained high but stable during the last 4 h. Baseline cortisol values, which recovered 1 h after animals were returned to family cages, were similar at the three different ages.

At 12 months of age, male and female marmosets showed a similar response, and a decrease in cortisol and below-baseline levels was observed in the two last phases, with statistical significance for females and statistical tendency for males. Buffering of the cortisol response to stressors around pubertal period was also observed in other studies using this animal model (49). Sachser et al. (50) argue that the adaptive value of this buffering supports migratory behavior, especially in females, which has been recorded from early adulthood in a number of species, such as occurs for common marmosets where females are the main sex to emigrate (42).

In Experiment III, immature marmoset males living in FGs throughout the study showed no statistically significant variation in cortisol levels across the phases. The non-significant, but important, increase in cortisol levels observed in FG in W5b with respect to W5a was possibly due to acute (10–15 min) family separation during the period of habituation to the experimental apparatus in memory tests, which occurred six times over 3 days during w5b. Smaller oscillations in cortisol levels in the IG were likely due to social support, which attenuates routine daily challenges (51). Galvão-Coelho et al. (11) reported on the benefits of social support among adult common marmoset males during periods of crises, reducing cortisol changes caused by social stressors.

On the other hand, cortisol was significantly altered in juvenile common marmoset males isolated from FGs for 8–11 months compared with those living in natal groups. In the first week of isolation, marmosets showed an increase in fecal cortisol, followed by a progressive drop over the ensuing months, exhibiting lower than baseline levels at the end of the experiment, when these animals were in their FGs. Different theories attempt to explain HPA axis hyporeactivity to stress. Some studies report that hypocortisolism is an adaptive and defensive mechanism during episodes of chronic HPA axis activation (52–54). Badanes et al. (55) found that chronic buffering of HPA activity may promote vulnerability, especially in critical periods of ontogenetic development such as the juvenile stage, in addition to facilitating subsequent emergence of psychiatric disorders such as depression. Moreover, recent studies have also associated hypocortisolism with a number of mental disorders, such as atypical and bipolar depression (56).

In addition to cortisol measurement, after 4 months of social separation, the animals were submitted to working memory tests. Males from the IG showed a negative correlation between cortisol and hits and a positive correlation between cortisol and errors. These findings are supported by evidence of a biphasic modulation of stress and cortisol profile suggesting that an increase in cortisol might be contributing to the impairment on this type of memory in the prefrontal cortex (PFC), where working memory occurs (57). Additionally, negative correlations between cortisol and AI for both groups (FG and IG) indicate that increased cortisol reduced motivation to perform working memory tests. Marmosets living in FGs were briefly separated, for 45 min at most, to undergo memory (object and box) tests. The number of hits and interest in performing the tests were lower than in marmosets in chronic social isolation. The three main reasons for the FG performing worse and being less motivated than their IG counterparts in the present study are as follows: (i) acute stress provoked by separation during the tests themselves and/or by a residual effect resulting from acute separation in the preceding period of habituation, which appears to have been too short to reduce the stress response; (ii) the fact that stress situations may produce motivational impairment and that isolated animals are deprived of sensorial stimuli; and (iii) animals are more aware of changes in their environment.

This study was based on the methodological approach developed by Roberts and Wallis (31) (object) and Murai et al. (32) (box) for common marmosets, in which few experimental sessions were used. The overall poor performance of the two groups in both direct and mainly reverse tasks calls into question the protocol used in this study, since other experiments report different levels of training required to learn visual discrimination tasks. Yamazaki and Watanabe (58) demonstrated that adult common marmosets require 20–300 trials before learning tasks, whereas our study used a maximum of 15 attempts per day, over 3 days. Spinelli et al. (59) report that adult common marmosets usually commit around 50 errors before learning a direct task and about 100 before learning a reverse task. Moreover, with training, marmosets were able to perform visual discrimination tasks and learn reversal tasks in both automated and non-automated working memory tests (32, 60, 61). Thus, the limitations shown by the animals in the present study are likely due to the lower number of trials used in the protocol, as well as the stress induced by acute (isolation for habituation before tests) and/or chronic isolation, which poses an additional challenge, thereby reducing learning. Furthermore, studies suggest that species with cooperative reproductive systems, such as common marmosets, perform better in cognitive tasks involving social learning, such as communication, imitation, and cooperation, than in tasks using working memory, inhibitory control, and tools (62).

In summary, the findings of the present study showed that during common marmosets’ development, cortisol profile is variable, becoming dimorphic in males and females as early as 4 months of age. In both sexes, a cortisol decrease occurs at 3 months, with females showing a more acute decrease and recovery, to below birth levels, but higher than that of males through juvenile II and subadult ages. Cortisol in males and females was more changeable over 21 days of social isolation at 6 and 9 months, showing differences between males and females, although no typical sex pattern could be characterized. Cortisol decreased in both female and male marmosets at 12 months of age after 21 days of social isolation. As shown in juvenile males under chronic social isolation for 4 months, cortisol decline was sustained during this period and marmosets in this situation displayed better cognitive performance and motivation when compared to those submitted to short-term stress living in FGs. Thus, since cortisol profile seems to be sexually dimorphic early in life, age and sex are critical variables to consider in approaches that require immature marmosets in their experimental protocols. Moreover, the cognitive tests available should be scrutinized to allow better investigation of cognitive traits in this species.

## Author Contributions

MS and NG-C designed the experiments; MS and DC carried out statistical analysis of Experiment I; NG-C and AG carried out statistical analysis of Experiment II; NG-C and AG carried out statistical analysis of Experiment III; MS and AG prepared figures; DC collected experimental data from Experiment I; CS collected experimental data from Experiment II; AG collected experimental data from Experiment III; and MS, NG-C, and AG prepared the manuscript.

## Conflict of Interest Statement

The authors declare there is no conflict of interest that would compromise the independence of this work.
